# Comparing the emotional impact of the UK COVID-19 lockdown in very preterm and full-term born children: a longitudinal study

**DOI:** 10.3389/frcha.2023.1193258

**Published:** 2023-06-13

**Authors:** Zeyuan Sun, Laila Hadaya, Marguerite Leoni, Paola Dazzan, Emily Simonoff, Serena J. Counsell, A. David Edwards, Chiara Nosarti, Lucy Vanes

**Affiliations:** ^1^ Department of Child and Adolescent Psychiatry, Institute of Psychiatry, Psychology and Neuroscience, King’s College London, London, United Kingdom; ^2^ Centre for the Developing Brain, School of Biomedical Engineering & Imaging Sciences, King’s College London, London, United Kingdom; ^3^ Department of Psychological Medicine, Institute of Psychiatry, Psychology and Neuroscience, King’s College London, London, United Kingdom; ^4^ Department of Neuroimaging, Institute of Psychiatry, Psychology and Neuroscience, King’s College London, London, United Kingdom

**Keywords:** COVID-19, child mental health, very preterm, internalizing symptoms, lockdown, crisis

## Abstract

**Introduction:**

The COVID-19 pandemic has caused a global mental health crisis, especially for those individuals who are vulnerable to stress and anxiety due to pre-existing mental health problems. This study aimed to understand the emotional impact of the COVID-19 lockdown on children who were born very preterm (VPT, <32 weeks' gestation), as they are vulnerable to mental health difficulties and are at increased risk of developing psychiatric problems during childhood compared to their full-term-born counterparts.

**Methods:**

The parents of 32 VPT children (mean age = 8.7) and 29 term-born controls (mean age = 8.8), who had previously taken part in a study of brain development and psychopathology following VPT birth, completed an online modified version of the Coronavirus Health and Impact Survey (CRISIS). The emotional impact of the COVID-19 lockdown on the child and the parent, measured by the CRISIS, was studied in relation to pre-existing mental health, assessed with the parent-rated Strengths and Difficulties Questionnaire (SDQ), evaluated before the CRISIS completion (mean time gap 15 months). Linear regression model comparisons were conducted to study the effects of COVID-19-related stressors on children's and parents' behavior, relationships and mental health.

**Results:**

There were no significant group differences in pre-existing SDQ internalizing/externalizing symptoms, child's emotions or parent's emotions during the COVID-19 lockdown. However, higher pre-existing internalizing symptoms in VPT children were associated with greater lockdown-related emotional problems and worries (simple slope = 1.95, *p* < 0.001), whereas this was not observed in term-born children.

**Conclusion:**

Our results suggest that VPT children with pre-existing internalizing problems may be more vulnerable to the negative impact of certain societal and familial stressors, such as social restrictions during the national COVID-19 lockdown periods. Further rigorous studies are therefore needed to assess the severity of increased risks for this particularly vulnerable group in the context of potentially stressful life changes and adjustments.

## 1. Introduction

Restrictions to daily life during the COVID-19 pandemic had profound effects on children's well-being, friendships and mental abilities. Closure and reduction of access to academic settings and routine medical care resulted in decreased social support to children and young people, with likely adverse consequences for their mental health (1–3). In addition, the COVID-19 pandemic was associated with socioeconomic challenges for some families, due to increasing financial pressure, income decline and job loss (4, 5). Taken together, such factors contributed to changes in family dynamics during these uncertain times, in some instances exacerbating psychological stress for all family members (6).

Whilst it is now established that the COVID-19 pandemic has caused a global secondary mental health crisis (7, 8), this appears to be especially true for those individuals who are vulnerable to stress and anxiety due to pre-existing mental health conditions (9, 10). In uncertain times and when facing stressful events, such individuals may be particularly worried about what is happening, become socially isolated and, at the extreme end, experience mental health problems (11, 12).

Here we studied the emotional impact of the COVID-19 lockdown on children who were born very preterm (VPT, <32 weeks’ gestation) as they are vulnerable to stress and anxiety (13). Furthermore, VPT children have also been found to show a more than twofold incidence of anxiety symptoms in the clinical range compared to their full-term born peers (14, 15), to experience increased emotional and behavioral symptoms in young adult life (16) and to be at increased risk of receiving a diagnosis of attention-deficit/hyperactivity disorder (ADHD) (17). VPT children also have a doubled risk of developing clinically significant anxiety compared to full-term-born children (18).

Given the pre-existing vulnerability of VPT children to mental health difficulties, we investigated the effects of COVID-19-related stressors on children's and parents' behavior, relationships and mental health. We hypothesized that VPT children would be more negatively impacted than their term-born peers by lockdown-related stressors and that their pre-existing mental health would be associated with COVID-19 related emotional problems. Understanding the impact of COVID-19 on VPT children will help us understand what type of mental health support is needed, now and in the future.

## 2. Methods

### 2.1. Participants

This longitudinal study recruited parents of very preterm and full-term children who had taken part in the Brain, Immunity and Psychopathology following very Preterm birth (BIPP) study. The BIPP study is currently ongoing, inviting consenting participants who previously took part in the “Evaluation of MR imaging to predict neurodevelopmental impairment in the preterm infant” study [ePrime; EudraCT 2009-011602-42 (19)] to complete a follow-up assessment between the ages of 8 and 10 years. Eligible participants were those who had previously taken part in a behavioral follow-up assessment at the age of 4–7 (20, 21). Infants recruited into ePrime had the following inclusion criteria: birth before 33 weeks of gestation, maternal age above 16 years, and mothers not being hospital inpatients. Exclusion criteria were major congenital malformations, contraindications to magnetic resonance imaging, parents not being able to speak English, or being subject to child protection proceedings. 511 very preterm infants delivered at 14 hospitals in the North and South-West London Perinatal Network were recruited at birth between April 2010 and July 2013 (19).

Full-term (FT) born controls matched for sex and age are also currently being studied as part of the BIPP study. Controls were recruited via three strategies: asking parents of preterm children to invite a child of the same sex and similar in age within the same academic year to participate in the study, through recruitment letters to local schools and via internal advertisements to college staff and students. Inclusion criteria are full-term birth (38–42 weeks) and birth weight >2,500 grams, the exclusion criteria are a history of neurological conditions (meningitis, head injury and cerebral infections) and contraindication for MRI. The BIPP study aims to recruit 240 VPT and 120 term-born participants by August 2024.

**Figure 1:**
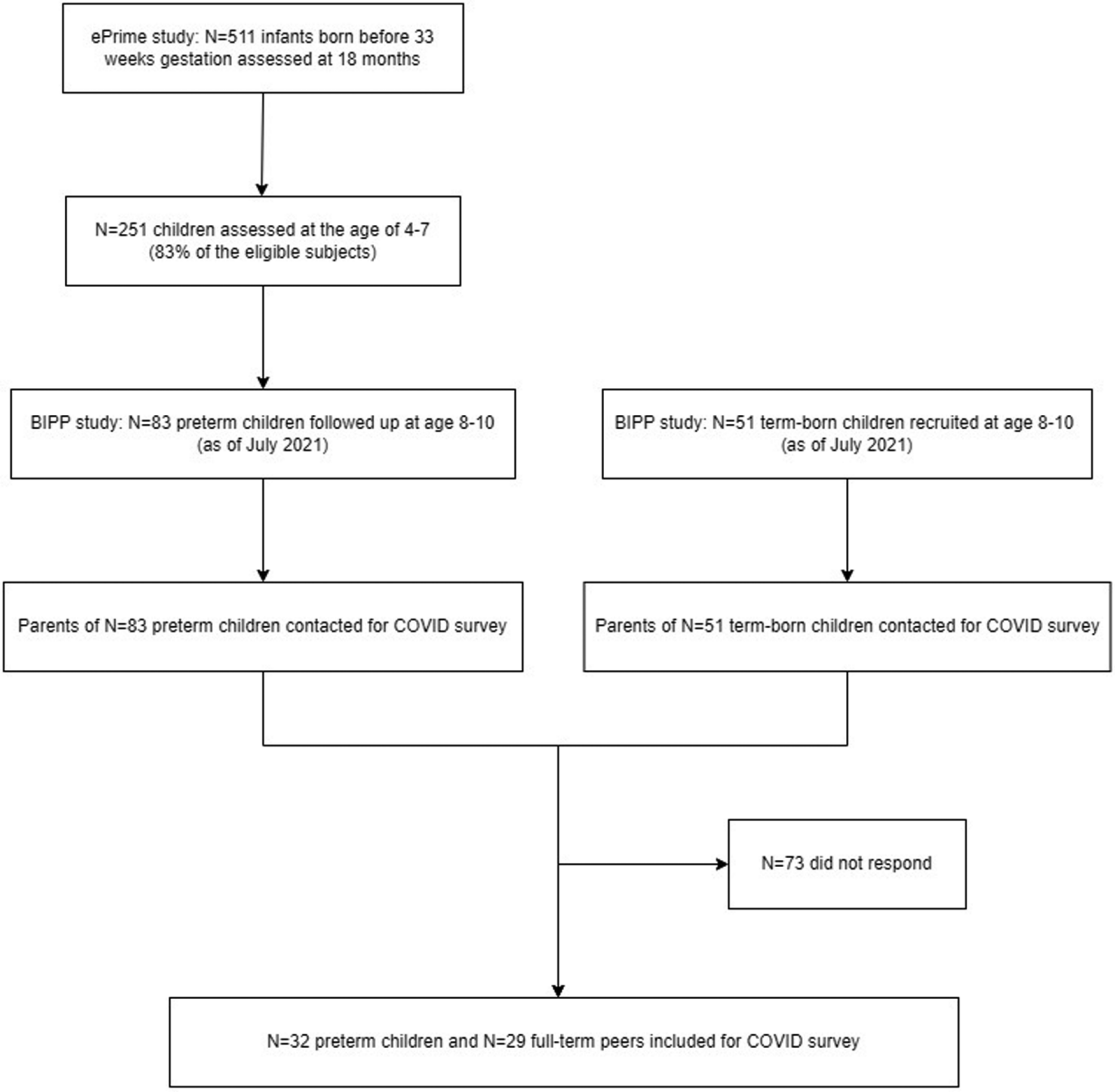
Flow chart of participants inclusion. For the follow-up at 4–7 years of age, a convenience sample (*N* = 251) was recruited corresponding to 82% of 306 participants who were past their fourth birthday by the follow-up study end date (September 1st, 2019), and had consented to be contacted for future research.

The parents of 134 BIPP participants (83 VPT children and 51 FT) who had already been assessed in person between October 2018 and July 2021 were contacted via email in September 2021 and asked to complete an online modified version of the Coronavirus Health and Impact Survey (CRISIS) (22). The current study included 32 questionnaires completed by a parent of a VPT child and 29 questionnaires completed by a parent of a term-born child (Figure 1).

### 2.2. Assessments

#### 2.2.1. Coronavirus Health and Impact Survey (CRISIS)

The Online Surveys platform (https://www.onlinesurveys.ac.uk) was used to obtain participants' informed consent and complete the CRISIS. The survey was completed by one of the child's legal guardians (97% birth mothers, 3% birth fathers). All the other assessments had already been collected in person as part of the BIPP study prior to the CRISIS completion between October 2018 and July 2021.

The CRISIS was created to assess the mental health impact of the COVID-19 pandemic, covering key domains relevant to mental distress and resilience (22). All three versions (for adults, parents/caregivers and youth) cover six domains, including COVID-19 exposure, COVID-19-related emotions/worries, life changes, mood states, substance use and daily behaviors. Our online survey used the parent/caregiver form to assess the impact of COVID-19 on children, and the adult self-report form to assess its impact on the responding parent (V1.0, http://www.crisissurvey.org/download/). Questions were rephrased to reflect the time during the UK's government-imposed COVID-19 lockdown period, rather than focusing only on the past two weeks, as in the original version. The varying degrees of national restrictions in the United Kingdom ranged from forced “stay at home measures” to eased “2m rules” and “Rule of six”.

For this study, we focused only on items in the “Emotions/Worries” sections pertaining to either the child or the parent (see Table 1 and Appendix). Responses to 13 items in the Emotions/Worries section of the parent/caregiver form were coded as 0 to 4 and the sum of scores was calculated to derive a continuous “child emotions” variable. The same coding was used for 10 questions in the “Emotions/Worries” section of the adult self-report form, and the sum of scores was calculated as a continuous variable to reflect parent emotions. Internal reliability of both the child and parent emotions subscales were found to be acceptable in our sample, with Cronbach's alpha for the child emotions subscale of 0.828, 95% CI: [0.747, 0.880]; and for the parent emotions subscale of 0.735, 95% CI: [0.622, 0.804].

**Table 1:** Adapted CRISIS questions for each item included in the primary outcome variables (child's emotions and parent's emotions).

Variable	Item
Child's emotions/worries (during the lockdown period):	1. How worried was your child generally?
	2. How happy vs. sad was your child?
	3. How much was your child able to enjoy his/her usual activities?
	4. How relaxed vs. anxious was your child?
	5. How fidgety or restless was your child?
	6. How fatigued or tired was your child?
	7. For their age, how well has your child been able to concentrate or focus?
	8. How irritable or easily angered was your child?
	9. How physically aggressive towards others was your child?
	10. How physically aggressive have others been towards your child?
	11. How lonely was your child?
	12. How worried was your child about being infected?
	13. How worried was your child about friends or family being infected?
Parent's emotions/worries (during the lockdown period):	1. How worried were you generally?
	2. How happy vs. sad were you?
	3. How much were you able to enjoy your usual activities?
	4. How relaxed vs. anxious were you?
	5. How fidgety or restless were you?
	6. How fatigued or tired were you?
	7. How well were you able to concentrate or focus?
	8. How irritable or easily angered were you?
	9. How lonely were you?
	10. How worried were you that you or someone in your family would become infected?

#### 2.2.2. Strengths and difficulties questionnaire (SDQ)

Parents had previously completed the Strengths and Difficulties Questionnaire (SDQ) (23, 24) as part of the BIPP study. The SDQ is a 25-item questionnaire to assess behavioral and emotional symptoms used to evaluate mental health concerns in children and young people aged 4–17. The SDQ comprises five sub-scales of five items each: emotional symptoms; conduct problems, hyperactivity/inattention, peer relationship problems and prosocial behavior. For this study, the “Emotional Symptoms” and “Peer relationship” subscales were combined into an internalizing subscale, while the “Conduct problems” and “Hyperactivity/inattention” subscales were combined into an externalizing subscale (24).

Internalizing and externalizing subscales were considered to reflect pre-existing mental health in the children. However, given the between-participant variation in the amount of time elapsing between completion of the SDQ and the CRISIS, a time gap variable was calculated as the number of days between the SDQ assessments and CRISIS survey completion, which was used in all further analyses as a covariate of interest.

#### 2.2.3. Wechsler intelligence scale for children, fourth edition (WISC-IV)

The Wechsler Intelligence Scale for Children, Fourth Edition (WISC-IV) (25) was also administered as part of the BIPP study. Full-scale intelligence quotient (IQ) scores were derived as a measure of children's cognitive abilities.

### 2.3. Statistical analysis

Statistical analyses were performed in R-4.2.1 and RStudio-1.4.1717. Independent samples t-tests were used to probe differences between the VPT and the full-term (FT) groups on continuous variables of interest. Separate linear regression models were run to test for the effects of pre-existing internalizing symptoms (or externalizing symptoms, respectively) and group (VPT vs. FT) on children's emotions during the lockdown. These regression models controlled for sex, age, time interval (between SDQ and CRISIS assessments), and parent's emotions during the lockdown.

Each model was compared to a further model including the interaction between internalizing (or externalizing, respectively) and group using likelihood ratio *F*-tests, to determine whether the association between pre-existing mental health symptoms and children's emotions differed between groups. In the case of a significant interaction, simple slope analyses were conducted to quantify the effect. Due to group differences in IQ, we also reran these regression analyses after inclusion of IQ as an additional covariate of no interest in all models.

## 3. Results

### 3.1. Sample characteristics

Table 2 presents the characteristics of the study sample. There were more boys than girls in the VPT group and more girls than boys in the control group, but there was no group difference in age, internalizing, or externalizing symptoms. FT children had significantly higher IQ than VPT children. There were no significant differences between participants included in this study and the overall BIPP sample at time of study in terms of SDQ internalizing symptoms, externalizing symptoms, age, or sex distribution, all *p*s > .05.

**Table 2:** Demographic, clinical, cognitive and pre-lockdown mental health characteristics of the study participants.

	Variable	Full-term (*n* = 29)	Preterm (*n* = 32)	Statistics
Demographic and clinical measures		**N(%)**	**N(%)**	**Chi-Square**
	Sex			
	Male	8 (27.6)	20 (62.5)	*X*^2^ = 6.13*
	Female	21 (72.4)	12 (37.5)	
		**Mean (SD)**	**Mean (SD)**	***t* (95% CI)**
	Age	8.8 (0.8)	8.7 (0.7)	0.58 (−0.27, 0.49)
	Gestational Weeks	39.9 (1.2)	29.8 (2.3)	22.08 (9.14, 10.97)***
	IQ	112.1 (12.5)	104.0 (16.0)	2.19 (0.70, 15.45)*
Pre-lockdown measures	SDQ Externalising	4.9 (2.9)	6.3 (3.7)	−1.66 (−3.05, 0.28)
	SDQ Internalising	4.7 (3.0)	5.9 (2.7)	−1.57 (−2.62, 0.32)

SD, standard deviation; SDQ, Strengths and Difficulties Questionnaire.

* *p* < 0.05, **p < 0.01, ***p < 0.001.

### 3.2. COVID-19 related child and parent emotions

In order to explore differences between groups in COVID-19 related child and parent emotions (indexed by the CRISIS), univariate linear regressions were conducted with group, sex, age and time gap as predictors. There was no difference in COVID-19 related child emotions between the VPT (*M* = 16.97, SD = 9.16) and control (*M* = 15.66, SD = 5.84) groups, *B* = 0.77 [−3.71, 5.25], *p* = 0.73; and no difference in parent emotions between the VPT (*M* = 19.75, SD = 6.48) and control (*M* = 19.13, SD = 6.17) groups, *B* = −0.10 [−184, 3.63, 3.41], *p* = 0.95, after accounting for the aforementioned confounders.

### 3.3. Pre-existing internalizing and externalizing symptoms and COVID-19 related child emotions

**Figure 2:**
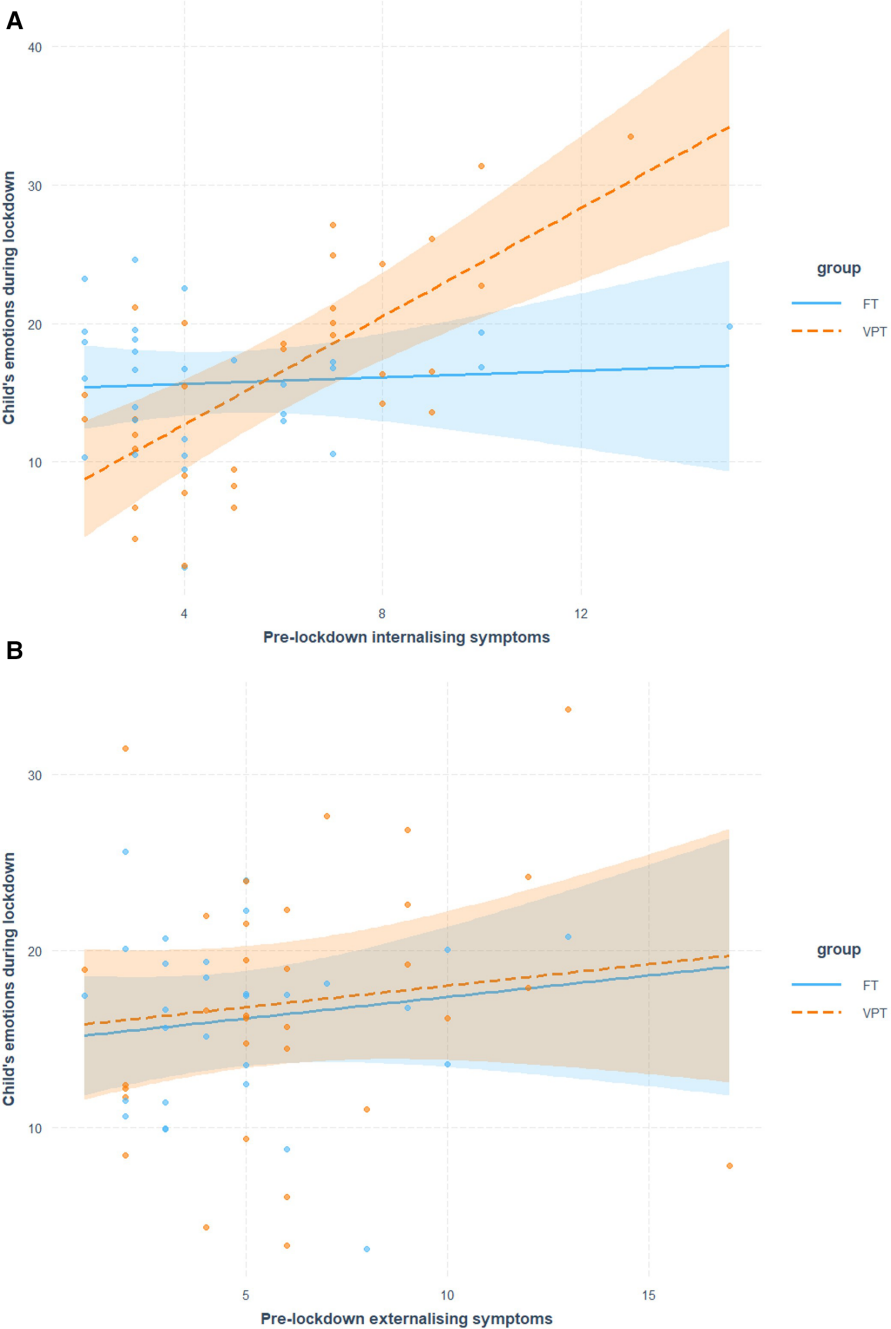
Scatter linear regression plot describing associations between pre-existing internalizing (**A**) and externalizing (**B**) symptoms and emotions during lockdown in VPT and FT children.

A model comparison via likelihood ratio *F*-test demonstrated that the model predicting child emotions during the lockdown from pre-existing internalizing symptoms (and adjusting for age, sex, time gap, and parent's emotions) was significantly improved by the inclusion of an interaction between group and pre-existing internalizing symptoms (*F* = 13.09, *p* < 0.001). The results of this model are shown in Table 3 (Model 1) and Figure 2A. A simple slope analysis revealed that, while in FT children there was no significant association between pre-existing internalizing symptoms and lockdown-related emotional problems (simple slope = 0.12, *p* = 0.74), VPT children showed a significant positive association between the two (simple slope = 1.95, *p* < 0.001), suggesting that higher pre-existing internalizing symptoms were associated with greater emotional problems and worries during the COVID-19 lockdown. Interestingly, after including these effects of pre-existing internalizing symptoms and their interaction with group, the main effect of group on COVID-19 related child emotions also became significant, indicating increased emotional problems in VPT compared to FT children (see Table 3) when taking internalizing problems into account. Inclusion of IQ in both the simple and interaction model did not alter the results of the model comparison (*F* = 12.89, *p* < 0.001).

**Table 3:** COVID-19 related child's emotions model predictors.

Dependent variable: Child's emotions during lockdown		
Model 1 predictors	*B* [95% CI]	*p*-value
Group: VPT	−10.28 [−16.53, −4.04]	0.002
Age	0.64 [−1.64, 2.92]	0.575
Sex: Male	0.63 [−2.63, 3.88]	0.702
Time gap	0.00 [−0.00, 0.00]	0.413
Pre-lockdown internalizing symptoms	0.12 [−0.59, 0.83]	0.740
Parent's emotions during lockdown	0.59 [0.35, 0.83]	<0.001
Interaction of group and internalizing symptoms	1.84 [0.81, 2.86]	<0.001
Model 2 Predictors	B [95% CI]	*p*-value
Group: VPT	0.63 [−3.15, 4.42]	0.346
Age	1.46 [−1.28, 4.21]	0.291
Sex: Male	−0.66 [−4.61, 3.27]	0.736
Time gap	0.00 [−0.00, 0.01]	0.223
Pre-lockdown externalizing symptoms	0.24 [−0.31, 0.79]	0.378
Parent's emotions during lockdown	0.69 [0.40, 0.98]	<0.001

Another model comparison using the likelihood ratio *F*-test demonstrated that the fully adjusted model (age, sex, time gap, and parent emotions) predicting child emotions during the lockdown from pre-existing externalizing symptoms was not significantly improved by the inclusion of interaction between group and pre-existing externalizing symptoms (*F* = 17.97, *p* = 0.53). The results of this model are shown in Table 3 (Model 2) and Figure 2B. Results suggest that there was no association between externalizing symptoms and emotional problems and worries during the COVID-19 lockdown in either VPT or FT children. Inclusion of IQ in both the simple and interaction model did not alter the results of the model comparison (*F* = 0.24, *p* = 0.63).

## 4. Discussion

Results of this study indicate that the emotional impact of the COVID-19 lockdown did not differ between VPT children and their term-born peers as a whole; they also show comparable effects of lockdown-related stressors on emotions and worries of the parents of VPT and full-term children. However, results of this study indicate that specifically among VPT children, higher pre-existing internalizing symptoms were associated with more COVID-19 related emotional problems and concerns during the lockdown. Importantly, these findings controlled for key demographic variables as well as the parents' own lockdown-related emotions and worries. The latter was indeed found to be significantly associated with children's emotions, which likely reflects both shared familial effects of the lockdown on parent and child, as well as potential rater bias given that all scales were completed by the parent.

Our findings are in line with a recent longitudinal study which showed that preterm birth and pre-existing mental health problems were associated with a greater risk for emotional and attention-deficit/hyperactivity disorder symptoms during lockdown (26). Another study comparing the impact of the COVID-19 lockdown on three groups of children found that the lockdown had a substantial influence on the entire family and added stress to families with children who were at risk for neurodevelopmental deficits (6). Evidence from two British cohorts also suggested that children with autism and their parents, who had experienced more pre-pandemic mental health symptoms, were more likely to have more pandemic-related mental health symptoms (27).

In terms of the association between pre-existing psychiatric risk and the emotional impact of national lockdowns, findings to date have been inconsistent. A recent study indicated that the emotional impact of COVID-19 was not exacerbated in children with early brain injury or low IQ (28); another study showed a detrimental impact of lockdowns on mental well-being only in young people without pre-existing depressive symptoms (29). However, other studies found that the lockdown had severely increased pre-existing stress and depression (30, 31), suggesting there is substantial heterogeneity in COVID-19 related emotional impact across different populations (2, 9, 10, 32, 33). Our findings suggest that preterm children with pre-existing psychopathology represent a particularly vulnerable group in this context.

This study has several limitations. Firstly, the CRISIS questionnaire was administered only once and probed parents' and children's emotion during the course of COVID-19 lockdown, thus preventing a detailed evaluation of the timing and trajectories of lockdown effects on mental health. Some studies have in fact described gradually increasing symptom severity at the beginning of lockdown, which decreased after the lockdown ended (34, 35), while others suggested that the most severe mental health symptoms occurred in the early stages of lockdown, but declined fairly rapidly afterwards (36). Secondly, our sample size is relatively small for both groups, and findings therefore may not be generalizable to all VPT and term children; however, the studied sample did not significantly differ from the overall sample in terms of key characteristics such as age, sex, and psychopathology (37). Our study is also limited by the non-random sampling method for term-born peers, although this approach may ensure greater similarity between control and preterm participants (38). Thirdly, all assessments relied on parent-report, which could have led to measurement bias, although we included parents' emotional problems as a confounding variable in our analyses to control for this. Finally, as our findings relate to a UK-based sample, their generalizability to other countries may be limited, given substantial differences in relevant variables such as healthcare or severity of nationally imposed COVID-related restrictions.

## 5. Conclusion

This study demonstrates that internalizing problems were associated with greater susceptibility to a negative emotional impact of the COVID-19 lockdown in VPT, but not term-born children. Our results suggest that VPT children with pre-existing internalizing problems may be more vulnerable to the negative impact of certain societal and familial stressors, such as social restrictions during the national COVID-19 lockdown periods. Further rigorous work is required to assess the severity of increased risks for this particularly vulnerable group in the context of potentially stressful life changes and adjustments.

## Data Availability

The raw data supporting the conclusions of this article will be made available by the authors, without undue reservation.

## Appendix

**Appendix Table 1:** Adapted CRISIS questions and scoring for each item included in the primary outcome variables (child's emotions and parent's emotions).

Variable	Question/item	Scale
Child emotions/worries (during the lockdown period):	How worried was your child generally?	0 = Not worried at all 1 = Slightly worried 2 = Moderately worried 3 = Very worried 4 = Extremely worried
	How happy vs. sad was your child?	0 = Very happy/cheerful 1 = Moderately happy/cheerful 2 = Neutral 3 = Moderately sad/depressed/unhappy 4 = Very sad/depressed/unhappy
	How much was your child able to enjoy his/her usual activities?	0 = Extremely 1 = Very 2 = Moderately 3 = Slightly 4 = Not at all
	How relaxed vs. anxious was your child?	0 = Very relaxed/calm 1 = Moderately relaxed/calm 2 = Neutral 3 = Moderately nervous/anxious 4 = Very nervous/anxious
	How fidgety or restless was your child?	0 = Not fatigue or tired at all 1 = Slightly fatigue or tired 2 = Moderately fatigue or tired 3 = Very fatigue or tired 4 = Extremely fatigue or tired
	How fatigued or tired was your child?	0 = Not fatigue or tired at all 1 = Slightly fatigue or tired 2 = Moderately fatigue or tired 3 = Very fatigue or tired 4 = Extremely fatigue or tired
	For their age, how well has your child been able to concentrate or focus?	0 = Very focused/attentive 1 = Moderately focused/attentive 2 = Neutral 3 = Moderately unfocused/distracted 4 = Very unfocused/distracted
	How irritable or easily angered was your child?	0 = Not irritable or easily angered at all 1 = Slightly irritable or easily angered 2 = Moderately irritable or easily angered 3 = Very irritable or easily angered 4 = Extremely irritable or easily angered
	How physically aggressive towards others was your child?	1 = No aggression at all 2 = A little aggression, once or twice and not severe 3 = Some aggression but not severe 4 = Some aggression, including hurting your child 5 = Frequent aggression, including hurting your child
	How physically aggressive have others been towards your child?	0 = No aggression at all 1 = A little aggression, once or twice and not severe 2 = Some aggression but not severe 3 = Some aggression, including hurting your child 4 = Frequent aggression, including hurting your child
	How lonely was your child?	0 = Not lonely at all 1 = Slightly lonely 2 = Moderately lonely 3 = Very lonely 4 = Extremely lonely
	How worried was your child about being infected?	0 = Not worried at all 1 = Slightly worried 2 = Moderately worried 3 = Very worried 4 = Extremely worried
	How worried was your child about friends or family being infected?	0 = Not worried at all 1 = Slightly worried 2 = Moderately worried 3 = Very worried 4 = Extremely worried
Parent's emotions/worries (during the lockdown period):	How worried were you generally?	0 = Not worried at all 1 = Slightly worried 2 = Moderately worried 3 = Very worried 4 = Extremely worried
	How happy vs. sad were you?	0 = Very happy/cheerful 1 = Moderately happy/cheerful 2 = Neutral 3 = Moderately sad/depressed/unhappy 4 = Very sad/depressed/unhappy
	How much were you able to enjoy your usual activities?	0 = Extremely 1 = Very 2 = Moderately 3 = Slightly 4 = Not at all
	How relaxed vs. anxious were you?	0 = Very relaxed/calm 1 = Moderately relaxed/calm 2 = Neutral 3 = Moderately nervous/anxious 4 = Very nervous/anxious
	How fidgety or restless were you?	0 = Not restless at all 1 = Slightly restless 2 = Moderately restless 3 = Very restless 4 = Extremely restless
	How fatigued or tired were you?	0 = Not fatigue or tired at all 1 = Slightly fatigue or tired 2 = Moderately fatigue or tired 3 = Very fatigue or tired 4 = Extremely fatigue or tired
	How well were you able to concentrate or focus?	0 = Very focused/attentive 1 = Moderately focused/attentive 2 = Neutral 3 = Moderately unfocused/distracted 4 = Very unfocused/distracted
	How irritable or easily angered were you?	0 = Not irritable or easily angered at all 1 = Slightly irritable or easily angered 2 = Moderately irritable or easily angered 3 = Very irritable or easily angered 4 = Extremely irritable or easily angered
	How lonely were you?	0 = Not lonely at all 1 = Slightly lonely 2 = Moderately lonely 3 = Very lonely 4 = Extremely lonely
	How worried were you that you or someone in your family would become infected?	0 = Not worried at all 1 = Slightly worried 2 = Moderately worried 3 = Very worried 4 = Extremely worried
